# Effect of Current Waveforms during Directed Energy Deposition of 4043 Aluminum Alloy on Microstructure, Hardness, and Wear of Alloy

**DOI:** 10.3390/ma16206716

**Published:** 2023-10-16

**Authors:** Kumar Ujjwal, Katipalli Anand Kumar, Mukul Anand, R. K. Singh Raman, Alok Kumar Das

**Affiliations:** 1 Department of Mechanical Engineering, Indian Institute of Technology (ISM), Dhanbad 826004, Jharkhand, India; ujjwal.19dr0076@mech.iitism.ac.in (K.U.); 21mt0183@mech.iitism.ac.in (K.A.K.); mucoolanand@gmail.com (M.A.); 2 Department of Mechanical & Aerospace Engineering, Monash University, Clayton, VIC 3800, Australia; 3 Department of Chemical & Biological Engineering, Monash University, Clayton, VIC 3800, Australia

**Keywords:** wire arc additive manufacturing, aluminum alloy, waveforms, microstructure

## Abstract

Wire arc additive manufacturing (WAAM) was employed to fabricate 4043 aluminum alloy walls. To investigate the effects of sinusoidal, triangular, and rectangular waveforms of alternating current (AC) and their transients on the wall geometry, microstructure evolution, hardness, and wear properties were evaluated. The root mean square (RMS) current value was maximum for the rectangular and minimum for the triangular waveform. The section produced by the triangular waveform had the highest height-to-width ratio, indicating that this waveform can be a favorable choice for creating components using WAAM. The optical micrographs of the transverse cross-section of the printed sections revealed the grain structure produced with this waveform to be heterogeneous, having a columnar dendritic structure at the bottom and equiaxed at the top portion. The waveforms also had an impact on the hardness and wear characteristics of all the walls, which were attributed to their cooling rate.

## 1. Introduction

Additive manufacturing (AM) of aluminum alloys has gained significant attention in recent years owing to their widespread application due to their high specific strength and corrosion resistance [1,2]. The ASTM 52900 standard defines AM as “The process of joining materials to make objects from 3D model data, usually layer-upon-layer, as opposed to subtractive manufacturing methodologies” [3]. Wire arc additive manufacturing (WAAM) is one such process that utilizes continuous feeding of the wire into the arc to melt and deposit the material on the substrate plate or the formerly deposited layer [4]. The motion of the welding torch or the work table is controlled either by a computer-controlled CNC or robot [5]. WAAM’s high deposition rate and buy-to-fly ratio make it suitable for printing components with medium to low complexity [6]. It has been widely adopted in the automobile, aerospace, and marine industries. Due to its high reflectivity, aluminum is not easily processed by laser-based AM methods, which makes WAAM a more suitable option. In terms of arc welding techniques that utilize the electric arc, such as gas metal arc welding (GMAW), cold metal transfer (CMT), and gas tungsten arc welding (GTAW), GTAW-based WAAM stands out for its ability to deposit layers precisely with low surface waviness [7]. This is due to the arc stability of the electric arc and the comparatively slower deposition rate in GTAW-based WAAM [8,9]. However, mechanical anisotropy, distortion, residual stress, and porosity are the major challenges associated with WAAM [10].

The heat input during the WAAM process can be controlled by controlling the current waveforms. Kumar et al. [11] investigated how welding current waveforms (square, rectangular, triangular, and sinusoidal AC waveforms) influenced arc behavior, temperature variation, joint profile, and the formation of intermetallic compounds during gas tungsten arc welding (GTAW) of AA6061-T6 with steel. They also concluded that GTAW in the alternating current mode is suitable for joining quickly oxidizing materials like aluminum alloys. Mvola et al. [12] assessed the impact of welding current waveform and voltage on welded joints and examined several factors that govern the forces acting during metal transfer. Their findings indicated that certain specific factors, including welding current, voltage waveforms, and the direction of the electrode feeding, play a crucial role in enhancing the transfer of metal and heat from the electrode to the base metals. Additionally, they found that these variables significantly influence the geometry of the welded joint. Faria et al. [13] studied the impact of square waveform with alternating current on the geometry and aspect of welds, using 3% negative polarity. Miao et al. [14] studied the microstructure evolution and mechanical properties, including the grain morphology, phase compositions, elemental distribution, microhardness, and tensile properties of laser-assisted WAAM vis-à-vis common WAAM, and the correlation between mechanical performance and microstructure was analyzed. Kohler et al. [15] compared the effects of the welding process parameters (e.g., deposit direction, bead geometry, and molten pool length) on welds between 4047 and 5356 aluminum alloys produced using the CMT-WAAM process. The effects of process adjustments, such as arc length correction and pulse correction, on build-up quality were also examined. Mechanical properties like tensile strength, hardness, porosity, and residual stresses were analyzed using X-ray diffraction. Nie et al. [16] conducted a study on 4043 aluminum alloy components produced through the CMT-WAAM process. Wang et al. varied the electrode positive (EP) to electrode negative (EN) ratio and investigated the electrical waveforms during the fabrication of the Al–Zn–Mg–Cu alloy walls by CMT-based WAAM [17]. They determined the ideal EP/EN ratio, which yielded optimal forming accuracy and ensured stable deposition, to be 13:7. Zhang et al. [18] examined the metal transfer behavior and stability in the swing arc additive manufacturing of AZ91 magnesium alloy through the CMT process. They analyzed electrical waveforms, high-speed images of the weld pool, and forces acting on the droplet to achieve optimum parameters for stable deposition. Pradeep et al. [19] employed a data-driven methodology to create a finite element model to predict the thermal field during the WAAM process. Their study utilized real-time measurements of voltage and current values to model the heat source and achieved less than 1% error in predicting the peak temperatures of the thermal fields.

However, such an understanding of the effect of current waveforms on the components manufactured by the GTAW-based WAAM process is not available in the published literature. The current study investigates the effect of different current waveforms on the microstructure, hardness and wear of 4043 aluminum alloy sections developed by the GTAW-WAAM process.

## 2. Materials and Methods

**Figure 1:**
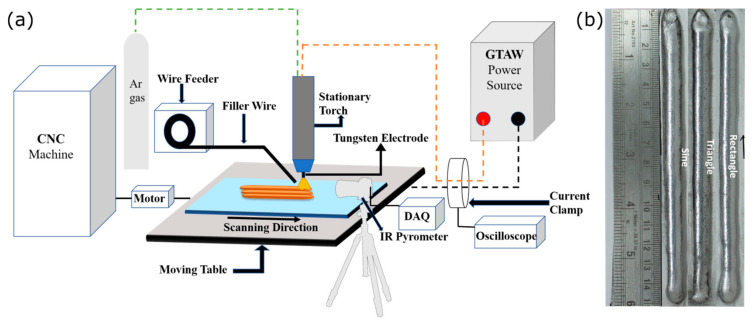
(**a**) Schematics of WAAM setup and (**b**) the deposited sections.

ER4043 aluminum alloy wire of 1.2 mm diameter and 6063 aluminum alloy substrate of thickness 4 mm was selected for the study. Aluminum alloy 4043 is commonly used as a filler wire in welding various series (1xxx, 2xxx, 3xxx, and 6xxx) of aluminum alloys [20]. This is primarily due to its exceptional fluidity and wettability. The choice of using 6063 aluminum alloy as a substrate is based on its compatibility with 4043 and its ability to facilitate epitaxial growth in the deposited layer [10]. Table 1 presents the chemical composition of the wire and the substrate. The GTAW-based WAAM setup consisted of a FRONIUS magic wave 2500 welding machine with the welding torch controlled by the computer numerical control (CNC) with SIEMENS 828D controller and 99.99% pure argon gas supply to create an inert environment during the WAAM process, as presented in Figure 1a. Five layers of the 4043 aluminum alloy were deposited utilizing three different waveforms (sinusoidal, triangular, rectangular), each 150 mm in length, as shown in Figure 1b. The waveforms were captured using a benchtop digital storage oscilloscope (Make: Tektronix, Beaverton, OR, USA; Model: TDS 2001C). The operating parameters for the deposited sections are described in Table 2. The infrared radiation (IR) pyrometer (Make: Micro-Epsilon, Raleigh, NC, USA; Model: CTLM-2H1SF300-C3) was used to capture the thermal cycle during the WAAM process.

The dimensions of the printed sections were measured at equal intervals using a Vernier caliper. Further, the sections were cut along the transverse direction and were hot-mounted before mechanical grinding. The mounted samples were subjected to mechanical grinding using papers up to 2000 grit, after which they underwent diamond polishing. The polished samples were etched with Keller’s reagent to reveal the grain boundaries, and microstructural features were observed under the optical microscope. The phases in the microstructure were identified using high-resolution X-ray diffraction (HR-XRD) (Make: Rigaku, Auburn Hills, MI, USA; Model: Smartlab), with 2θ varying from 20° to 100° at a scanning rate of 4°/min. The hardness of the deposited sections was measured using a Vickers hardness testing machine (Make: Mitutoyo, Kawasaki-shi, Japan; Model: HM220), as per ASTM E384 standards [21], at a load of 0.05 kgf and a dwell period of 10 s. A dry friction wear test at ambient temperature was carried out utilizing a universal tribometer (Make: Rtec Instruments, San Jose, CA, USA; Model: MFT 500), as per ASTM G133 standards [22], having a ball-on-flat configuration, which uses a 5 mm diameter SS 316 ball, with a stroke length of 5 mm at 10-Newton load for 10 min at 5 Hz frequency. The wear volume was calculated using a non-contact type profilometer (Make: Zygo, Middlefield, CT, USA; Model: Newview 9000).

## 3. Results and Discussion

### 3.1. Current Waveform

**Figure 2:**
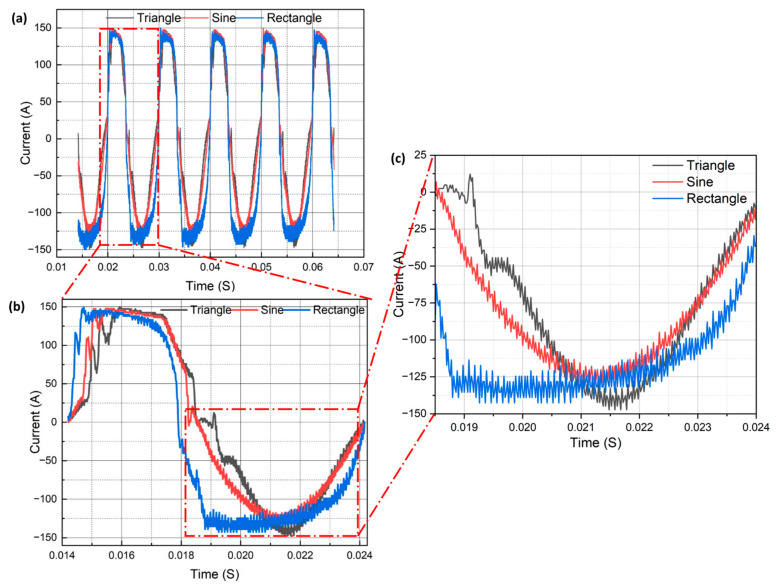
Acquired current waveforms (**a**) multiple cycles (**b**) single cycle (**c**) magnified image showing negative half cycle.

For welding and joining of aluminum-based metallic materials, alternating current (AC) is preferred as it aids in the removal of the tenacious oxide layer (cleaning action) formed on the surface [11]. Figure 2a depicts the current transients for the triangular, sinusoidal, and rectangular wave patterns. From the figure, it is evident that all the waveforms had the same wavelength and frequency. It can be seen in Figure 2b that the positive and negative half-cycles are asymmetric. This was due to the AC balance setting being set to a 40:60 ratio. This means that in a cycle, 40% of the time, the tungsten electrode will be positive and 60% time negative. This was conducted to facilitate more heat generation on the workpiece and less on the tungsten electrode. Each waveform had a peak current of 150 A during the positive half-cycle. However, for the negative half-cycle, the triangular waveform had a peak current of 150 A, the sinusoidal had a peak current of 131A, and the rectangular had a peak current of 142 A, as can be seen from Figure 2c. Also, the root mean square (RMS) current value for the rectangular waveform was 117 A, 102 A for sinusoidal, and 99 A for the triangular. This suggests that the heat input was maximum for the rectangular waveform and minimum for the triangular [23]. The corresponding voltage for each waveform can be calculated from the well-established relation V=10+0.04I [24], where V is the voltage and I is the current. However, the voltage was not monitored in this investigation. The rectangular waveform had the longest duration at peak current in the negative half-cycle, followed by the sinusoidal waveform and the triangular waveform (Figure 2b). This implies that the cleaning action is most prominent in the rectangular case, and more heat is generated on the workpiece than on the tungsten electrode.

### 3.2. Wall and Weld Bead Geometry

**Figure 3:**
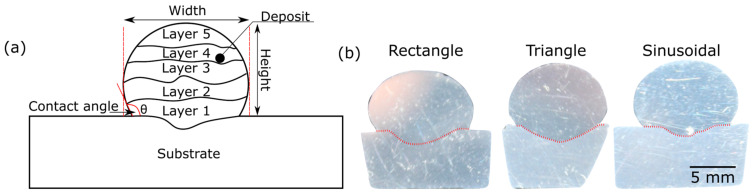
(**a**) Schematics of the deposited section, and (**b**) Cross-section of the deposited samples.

Figure 3a presents the schematic of the deposited section. For each layer, the scanning direction was kept the same. After the deposition of each layer, the welding torch was raised and repositioned to the start position for the subsequent layer deposition. The intention was to keep the time delay between the two layers to the minimum (about 30 s), thereby achieving a high interpass temperature. This high interpass temperature helps reduce porosity in the samples [25]. Though the deposited sections were of five layers, only three layers were visible as the top three layers remelted and fused together to form a single layer (Figure 3b). A similar behavior was reported by Bai et al. [26], where they found that the heat generated by GTAW was enough to remelt more than two layers. The width and height of all five-layered sections formed by different waveforms were measured, as presented in Table 3. The height was found to be maximum for the triangular and minimum for the rectangular waveform. In addition, the width was maximum for the rectangular and minimum for the triangular waveform, which is in line with their corresponding RMS current value. This behavior can be attributed to the longest duration of peak current in the negative half-cycle in the case of rectangular waveforms. Also, the height-to-width ratio for all the walls formed by different waveforms is summarized in Table 3. The five-layered wall’s height-to-width ratio was 0.97 for triangular, 0.84 for sinusoidal, and 0.76 for rectangular. This suggests that for the same wall height, the wall fabricated by the triangular waveform will have fewer layers than the other two. The contact angle was maximum for the rectangular and minimum for the sinusoidal waveform.

### 3.3. Cooling Rate

**Figure 4:**
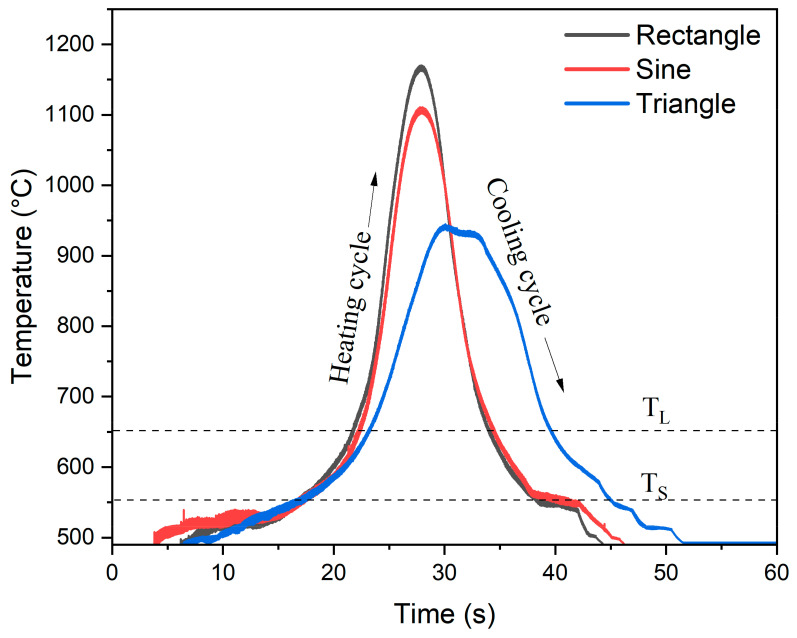
Temperature vs. time plots for different current waveforms.

The solidification behavior and microstructural evolution of alloys during welding processes can be better understood by analyzing the heating and cooling curves. The cooling curves play a vital role in optimizing process parameters and regulating the microstructure of the end product, specifically in the case of GTAW-based WAAM. The temperature of the substrate was monitored during the deposition process by employing an IR pyrometer. The measurement point was set at a distance of 5 mm away from the deposition zone. The temperature profile was obtained for the five-layered wall, the pyrometer readings were taken for every layer for each waveform, and their average was plotted, as illustrated in Figure 4. As the deposition started, the temperature of the measurement point on the substrate rose and attained a maximum as the electric arc approached the point (heating cycle). Subsequently, the temperature started to decrease as the electric arc moved past that point (cooling cycle). The T_S_ and T_L_ correspond to the solidus and liquidus lines for the 4043 Al-alloy [27]. The rectangular waveform had the maximum average peak temperature of 1164 ± 64 °C followed by sinusoidal (1049 ± 56 °C) and triangular (960 ± 38 °C). Also, the peak temperature for each waveform was much higher compared to the melting point of aluminum. This is attributed to the oxide formation on the wall, which required high temperatures for melting [28]. The average cooling rate was determined by averaging the slopes of the temperature–time curves in the cooling cycle region (between T_S_ and T_L_) at uniform intervals [29]. The calculated average cooling rate of the rectangle waveform was 23.3 ± 2 °C/s; for the sinusoidal waveform, it was 22.4 ± 1.5 °C/s, and for the triangle waveform, it was 17.75 ± 2 °C/s. The variation can be attributed to their corresponding RMS current value that contributed to the heat input, which is governed by the equation $H=\frac{\eta \times I\times V}{v}$, where *H* is the heat input per unit length, *η* is the heat transfer efficiency, *I* is the RMS current, *V* is the voltage, and v is the scanning speed [29].

### 3.4. Microstructure and X-ray Diffraction

**Figure 5:**
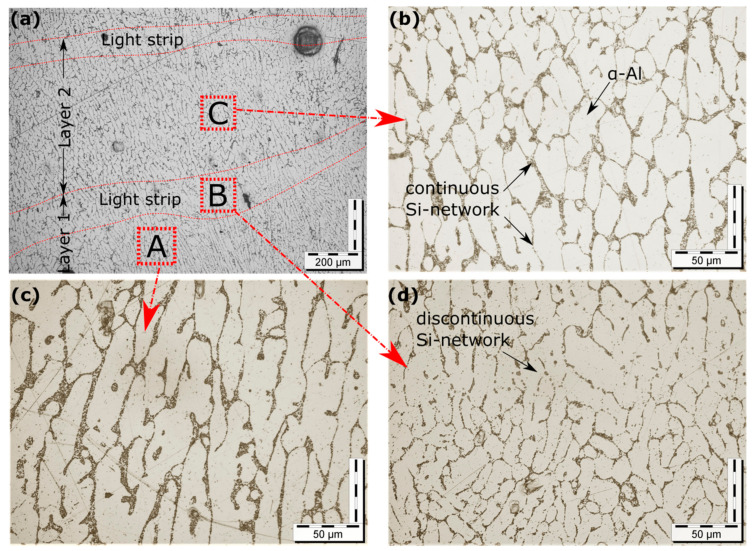
Microstructure of printed samples with different regions: (**a**) showing different layers separated by light strip, (**b**) magnified image of region C showing different phases present, (**c**) magnified image of region A showing columnar dendrites, and (**d**) magnified image of region B showing discontinuous Si-network.

**Figure 6:**
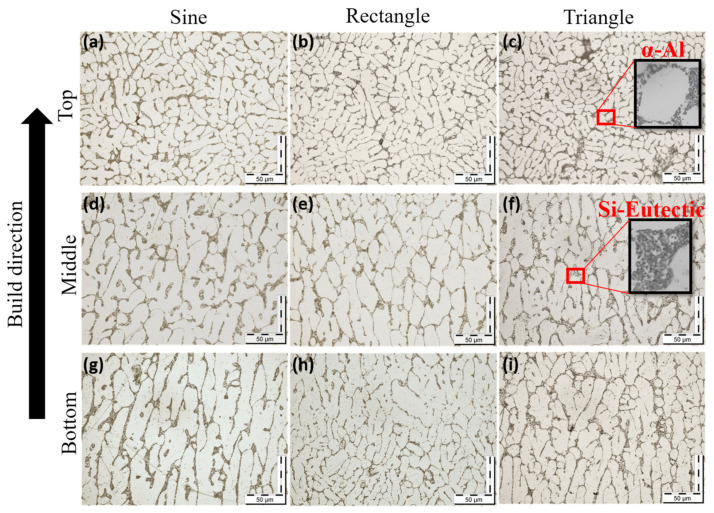
Optical micrographs of the transverse cross-section of different locations of the printed sample (**a**–**c**) top (**d**–**f**) middle (**g**–**i**) bottom.

**Figure 7:**
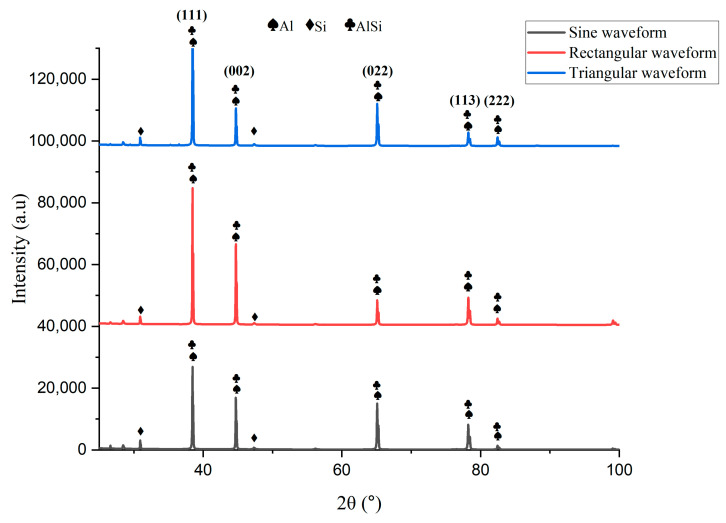
XRD plot of all the deposited sections.

The microstructure of the printed sample in the WAAM process is greatly influenced by process parameters like welding current, voltage, scanning speed, shielding gas flow rate, and interlayer delay time [29]. The size and distribution of the precipitates within the printed component are controlled by the cooling rate [30]. The micrographs of the printed sample depicting different regions are presented in Figure 5a–d. The microstructure primarily consisted of α-aluminum and aluminum-silicon eutectic [31], as shown in Figure 5b. This eutectic surrounds α-aluminum, forming a continuous silicon network. Figure 5a clearly shows that the deposited layers are separated by a distinct light strip. This light strip is formed as a result of the grain boundary diffusion, which causes the formation of a discontinuous silicon network during the deposition of subsequent layers [14], as presented in Figure 5d. Figure 6a–i shows the optical micrographs of the transverse sections of the five-layered deposits, depicting evolution in the microstructure in the build direction, with a coarser grain structure at the bottom, and fine at the top. This heterogeneous grain distribution can be attributed to the non-uniform temperature distribution in the build direction. The presence of the two phases was confirmed by the X-ray diffraction (XRD) data, as seen in Figure 7. In addition, there was the formation of Al_9_Si intermetallic, which is consistent with the findings reported by other authors [32]. The crystallographic growth of α-aluminum and α-aluminum-silicon eutectic occurs predominantly along (111) direction. The low intensity of the silicon-rich phase in the XRD spectra is attributed to the low Si content of the alloy. The 6063 aluminum alloy substrate assisted in the epitaxial growth of the first layer [10], as can be seen in the bottom section of all the samples presented in Figure 6g–i. The line intercept method was employed to measure the grain size of the transverse cross-section printed samples [33]. The shape and size of grains are influenced by the relationship between the temperature gradient (G) and the solidification rate (R). The G/R ratio governs the grain morphology, while the product of G and R determines the grain size [23]. The grain structure was predominantly columnar dendritic at the bottom portion, with the maximum average grain size of 80 ± 4 µm for the deposited section developed by rectangular waveforms, followed by sinusoidal (49 ± 2 µm) and triangular (45 ± 2 µm). The middle section exhibited a slightly smaller grain size than the bottom, with size ranging from (41 ± 4 µm) for rectangular waveforms to (39 ± 2 µm) for sinusoidal and (36 ± 2 µm) for triangular. At the same time, the top portion of all the deposited sections exhibited an equiaxed grain structure with an average grain size of nearly 25 ± 6 µm. A similar trend was reported by Lyu et al., which they attributed to the corresponding cooling rate [34].

### 3.5. Hardness and Wear

**Figure 8:**
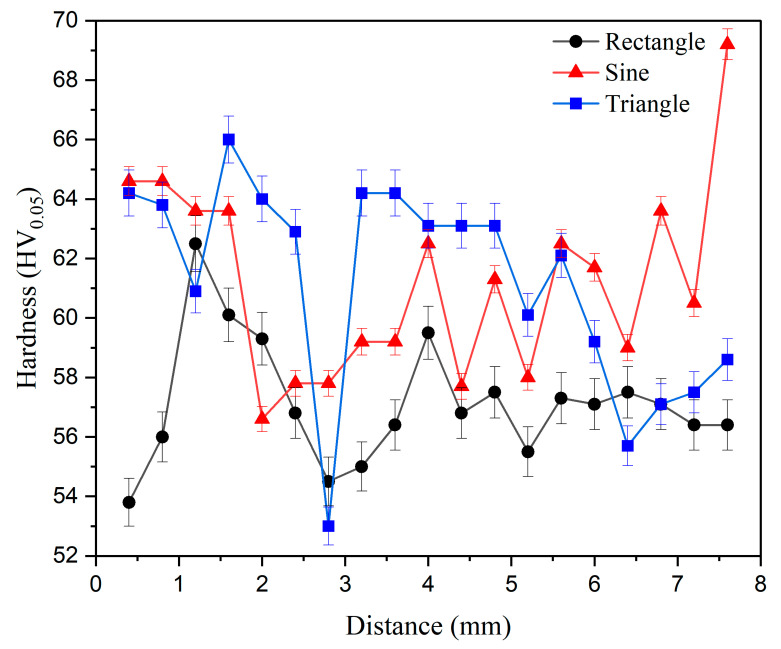
Vickers microhardness distribution for the different waveforms and its variation in moving from top to bottom.

Vickers microhardness test was carried out for all the deposits as per the standards [21], and its variation in moving from top to bottom is presented in Figure 8. The hardness was measured at an interval of 0.4 mm in moving from top to bottom (i.e., opposite to the build direction). There appears to be a pattern in the hardness of all the deposits, i.e., the values being somewhat higher at the top than those at the bottom, which is attributed to the difference in the average grain size at the top and bottom. Also, the erratic nature of hardness values depends on whether the indent is primarily on the α-aluminum or on the eutectic silicon phase; the latter has a higher hardness [35]. The average hardness value was maximum for triangular (63 ± 3 HV_0.05_) and minimum for rectangular (57 ± 2 HV_0.05_) waveform. This difference in hardness can be attributed to their respective average grain sizes. Since finer grains typically have a greater number of grain boundaries, this impedes the movement of dislocations more effectively than coarser grains [29].

**Figure 9:**
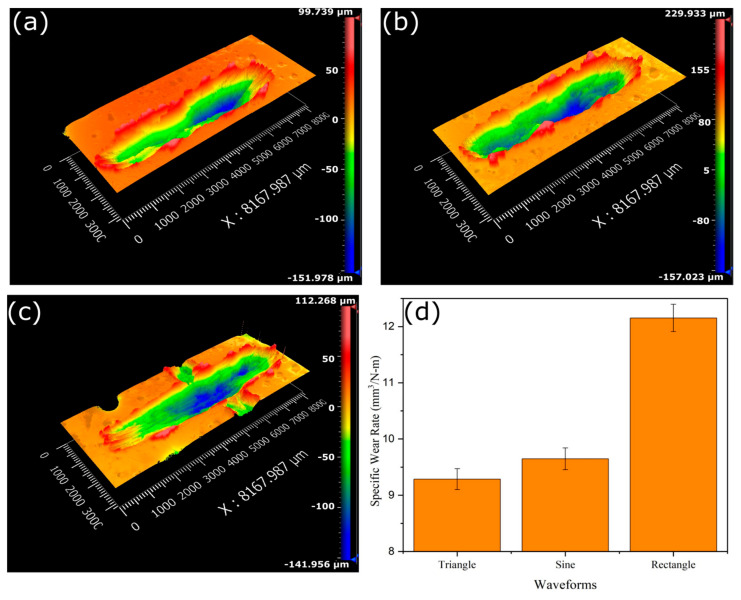
Surface profile of the worn surface: (**a**) sinusoidal, (**b**) rectangular, (**c**) triangular and (**d**) specific wear rate.

**Figure 10:**
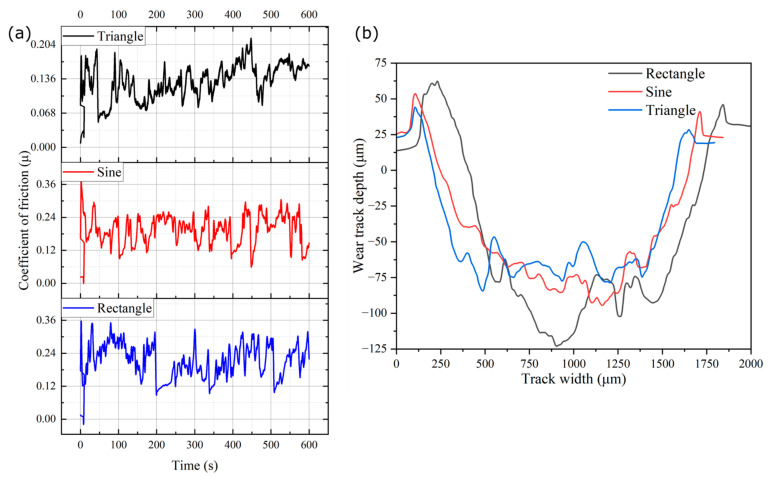
(**a**) Evolution of coefficient of friction (COF) for different samples and (**b**) wear-track cross-section profile.

**Figure 11:**
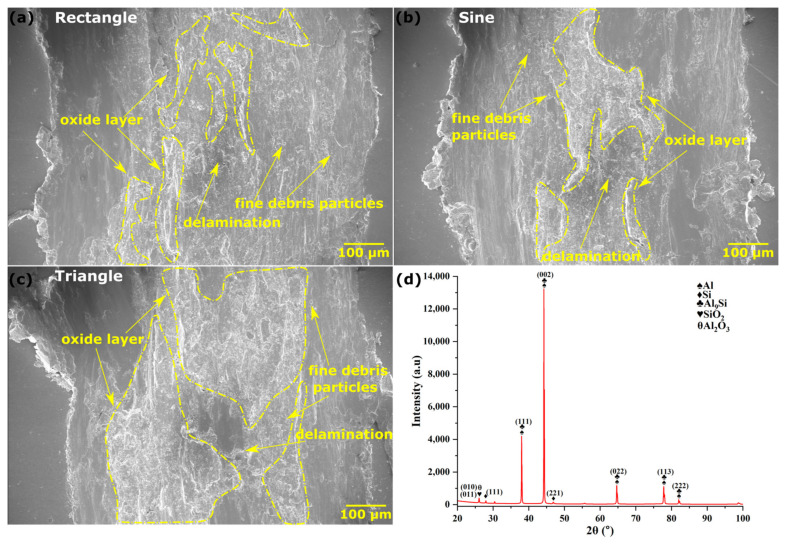
(**a**–**c**) SEM image of the wear track of rectangular, sinusoidal, and triangular wave patterns, respectively, and (**d**) XRD in the area of the wear track.

Figure 9a–c presents the surface profile of the worn-out surface after the wear test, and Figure 9d shows the specific wear rate data. The wear tests were performed on the cross-section of all the deposited sections as per the standards [22]. These cross-sections were mechanically ground/polished with emery paper (up to 2000 grit) before the wear test. The evolution of the coefficient of friction (COF) during the wear test is presented in Figure 10a. It can be seen from the figure that the COF is higher at the beginning, which can be ascribed to the higher static friction at the start of the test. The erratic nature of COF over time can be due to the repeated formation and breaking of cold weld joints at the interface. The average COF was calculated to be minimum for triangular (0.09 ± 0.03), followed by sinusoidal (0.13 ± 0.02), and maximum for rectangular (0.21 ± 0.03). The XRD in the area of the wear track (Figure 11d) revealed the presence of Al_2_O_3_ and SiO_2_ phases, confirming the presence of the hard oxide layer. The formation of this oxide layer gets enhanced due to the frictional heat generated during the wear test, which can reduce adhesive wear, subsequently resulting in a decrease in the COF [36,37].

The reason for this observed trend in the COF, with rectangular samples showing the highest value, followed by sinusoidal and triangular samples, can be attributed to their level of uniformity of the oxide layer. Figure 11a–c shows the oxide layer formed on the wear track of different samples. The oxide layer seems more continuous in the case of triangular, followed by sinusoidal and rectangular. The precise explanation for this behavior is unclear and requires further investigation. However, a similar trend in different materials has been reported by Mao et al. [38] and Wang et al. [39], where fine-grain structures have shown lower COF values than coarse grains. Wang et al. reported that the oxide layer formed on the wear track due to the friction heat cannot break easily in the case of fine grains as compared to those with coarse grains. As a result, the fine-grain structure exhibited low COF.

The wear track’s cross-section profile, as depicted in Figure 10b, clearly indicates that the samples fabricated using the rectangular waveform exhibited the deepest crater depth, while those produced with the triangular waveform had the shallowest crater depth. Archard’s equation was utilized to determine the specific wear rate [40]. From Figure 9d, it is evident that the specific wear rate is maximum for rectangular (12.2 ± 0.2 mm^3^/N-m) followed by sinusoidal (9.64 ± 0.19 mm^3^/N-m) and triangular (9.2 ± 0.18 mm^3^/N-m). This trend can be ascribed to the microhardness and average grain size of the samples. Samples with finer grain, which have more grain boundaries, tend to have higher hardness and wear resistance. This is because the presence of more grain boundaries provides greater hindrances to the movement of dislocations, leading to increased hardness and improved resistance to wear [41]. Figure 11a–c shows the SEM images of the wear tracks. The scattered debris of fine particles and delaminated layers on the wear track suggested that the mechanism for wear is predominantly adhesive and delamination [37].

The 4043 aluminum alloy series used in this study finds its application in the fabrication of the engine block, where the cylinder lining is subjected to wear due to the reciprocating action of the piston. Fabrication of the engine block through WAAM using the parameter corresponding to the minimum specific wear rate may enhance the wear resistance capability.

## 4. Conclusions

This study investigates the influence of different current waveforms on 4043 aluminum alloy sections fabricated by WAAM. The deposited sections were evaluated for their cross-sectional microstructure, XRD analysis, microhardness, and wear behavior. Based on the results obtained, the following conclusions can be drawn:

- The root mean square current was maximum for the rectangular waveform and minimum for the triangular, suggesting heat input to be maximum for the rectangular waveform.
- The higher height-to-width ratio for the section developed by the triangular waveform makes it more suitable for wall fabrication.
- The grain structure varies from columnar dendritic to equiaxed in the build direction, with the average grain size being maximum for rectangular and minimum for triangular.
- The specific wear rate was maximum for rectangular and minimum for triangular, which was attributed to the corresponding microhardness.

The analysis revealed that the choice of current waveform in GTAW-based WAAM could directly impact the key parameters, including heat input, wall geometry, grain structure, hardness, and wear behavior. These findings contribute to the broader understanding of the WAAM process and can facilitate advancement in research and industrial applications related to additive manufacturing of aluminum alloy.

## Figures and Tables

**Table 1:** Chemical composition (wt%) of wire and substrate.

Material	Al	Si	Fe	Cu	Mn	Mg	Zn	Cr	Ti
ER 4043	Bal	4.5–6	0.8	0.3	0.05	0.05	0.1	-	0.2
AA 6063	Bal	0.4	0.15	0.10	0.10	0.7	0.10	0.10	0–0.10

**Table 2:** Process parameters.

Current (A)	Voltage (V)	AC Frequency (Hz)	Scan Speed (mm/min)	Wire Feed Rate (m/min)	Gas Flow Rate (lt/min)
150	16	100	115	1.4	12.5

**Table 3:** Details of the five-layered section developed by different waveforms.

Waveforms ⮕ Dimensions ⬇	Rectangular	Sinusoidal	Triangular
Height (mm)	7.16 ± 0.35	7.73 ± 0.38	8.15 ± 0.4
Width (mm)	9.3 ± 0.46	9.2 ± 0.46	8.4 ± 0.41
Height-to-width	0.76	0.84	0.97
Contact angle (°)	132 ± 3	112 ± 4	126 ± 2

## Data Availability

Data are contained within the article.
